# Editorial Notes

**Published:** 1921-12

**Authors:** 


### Editorial Notes.

**The late Dr. Carey Pearce Coombs.**

We offer deep sympathy to Dr. Carey Coombs, our co-worker on the Editorial Staff, on the death of his distinguished father, Dr. Carey Pearce Coombs, J.P. Graduating as M.D. of London University in 1869, he settled in Castle Cary, where he enjoyed a very extensive practice and won for himself an influential position in the County of Somerset. As a physician he stood high in the estimation of his medical colleagues, who frequently sought his help in consultation in cases of difficulty. Many a time when attending meetings of the Bristol Medico-Chirurgical Society he made valuable contributions to our discussions. When, with mellowing years, the fragrance of simple courtesy and personal charm in the skilled practitioner, with long-life's knowledge of men, wins the trust and esteem of all who know him, he is "just one of the old school." Thus was Carey Pearce Coombs.

**American College of Surgeons.**

A complete file of the *Bristol Medico-Chirurgical Journal* from the year 1882 onwards, with the exception of two numbers (viz. 7 and 8 for the year 1885), has been presented by the Society to the new Reference Library of the American College of Surgeons. That the gift of our *Journal* has been warmly appreciated is evident from the courteous letter of acknowledgment received from the Secretary-General, who states that "This forms a very important addition to our Journal file, and I wish to extend to you on behalf of the Fellows of the American College of Surgeons our very sincere thanks and appreciation of your generous contribution. We hope very soon to build up a large reference library, and we felt that a complete file of your *Journal* was quite essential."

If any of our members can complete the set by presenting the two missing numbers mentioned above we shall be glad if he will kindly forward the same to the Assistant Editor.

**Bristol Medical Library.**

THIS letter from the American College of Surgeons reminds us of the advantages we enjoy in our own Medical Library. Sir William Osler, whose experience and judgment in the matter of Medical Libraries was great, had an almost envious admiration for the Bristol Medical Library, which he used to say was one of the best equipped in the provinces. The Medico-Chirurgical Society's library, conjoined with those of the Royal Infirmary and General Hospital, has been incorporated for many years with the Medical Library of the University, and together they comprise 24,629 books and 188 current periodicals.[1] Among the books many ancient master-works and rare editions are to be found ; but the principal value of a reference library lies in its periodicals. We are particularly fortunate in possessing an exceptional number of complete sets of periodicals both current and defunct. To those engaged in original research the importance of having access to such a mass of periodical literature cannot be overestimated. It is with no small pride that so modern a University as Bristol can point to its Medical Library, the foundations of which are coeval with that of the Royal

[1] 15,351 of the Medico-Chirurgical Society's books and 9,278 of the University's, and of the current periodicals 106 come through the Society and 82 through the University.

Infirmary and carry us back to the year 1735. Many of the books were in the possession of Bristol doctors far earlier than that date. It is true that the oldest medical book in Bristol (a Guy de Chauliac MS. *circa* 1424) is in the safe and jealous keeping of the City Reference Library, but its very existence in our city serves to recall the respectable antiquity of the traditions of medicine in the western capital.

The value of the Medico-Chirurgical Library in itself is considerable, for no less than £12,000 would be required to replace the books and journals alone. To a large extent our *Medico-Chirurgical Journal* has been the means of acquiring these modern books sent for review and the periodicals which are sent in exchange.

As examples of old and rare books belonging to the Society's Library we may mention : Paracelsus, *Chirurgia magna*, fol., Argentoratum, 1573 ; Galen (Claudius), *Opera omnia* (Greek text), 5 vols. (in 10), 1538 ; Vesalius, *De humani corporis fabrica*, fol. [1543] ; Fuchs (Leonhard), *De medendi methodo*, 1539 ; *Order (The) of the Hospitalls of K. Henry the VIII. and K. Edward the VI.*, 1557. [Black letter.]

**Prof. T. Loveday, Vice-Chancellor.**

To the newly-appointed Vice-Chancellor of the University of Bristol, Prof. T. Loveday, Principal of the University College of Southampton, we offer a cordial welcome.

**Dinner to Sir Isambard and Lady Owen.**

We look forward to the near occasion of the dinner in honour of Sir Isambard and Lady Owen, to be held at the Victoria Rooms, Clifton, at 7.45 p.m., on February 13th.

